# Size Estimation of Under-Reported Suicides and Suicide Attempts Using Network Scale up Method

**DOI:** 10.29252/beat-070202.

**Published:** 2019-04

**Authors:** Mehdi Moradinazar, Farid Najafi, Mohammad Reza Baneshi, Ali Akbar Haghdoost

**Affiliations:** 1 *Research Center for Environmental Determinants of Health (ECEDH), Public Health School, Kermanshah University of Medical Sciences, Kermanshah, Iran*; 2 *Modeling in Health Research Center, Institute for Future Studies in Health, Kerman University of Medical Science, Kerman, Iran*

**Keywords:** Network scale up, Suicide, Size estimation, Under reporting

## Abstract

**Objective:**

To estimate (under reporting) UR of SDS (Suicide deaths) and SAS (suicide attempts) in Kermanshah Province which is among provinces with high suicide rate in Iran.

**Methods:**

For estimating the size of UR suicide death registers, all cases of SAS and suicide deaths were retrieved from forensic medicine and health centers. Then, using network scale up method, a sample of 500 cases, aged 18 to 65 years, were randomly selected from the general population on the basis of age - sex proportion. To find the 95% confidence interval, bootstrap technique was used.

**Results:**

The average coverage of SDS was 58.4%, the lowest and highest coverage rate of SDS were attributed to self-immolation (34.2%) and hanging (81.2%), respectively. The coverage rate of SAS for self-immolation and deliberate self-poisoning were 82.4% and 77.2%, respectively. Size estimation of SAS by NSU method revealed that deliberate self-poisoning with medication (61.7%), poisoning with toxins and chemicals (20.6%), and self-immolation (7.7%) were the most frequent methods of SAS.

**Conclusion:**

Given the low coverage of suicide registers, all causes of death, especially deaths classified as accident or deaths with undetermined category, are required to be accurately registered. Investigations of causes of death, correction of wrong codes, as well as interviews with survivors to give them assurance can reduce the rate of suicide denial and result in increased accuracy of death register coverage.

## Introduction

Suicide is a psychical-social issue of many countries. Suicide has dedicated a significant part of primary and secondary health care to itself [1]. In Iran as well as many other countries in the world, suicide has always been condemned as a notorious act; however, annual statistics of people who end their life in this way are increasing [2]. According to the World Health Organization report in 2006, nearly a million people die of suicide every year which is more than the number those died in war or as a result of criminal acts. SAS is 20 times more. Nearly 75% of suicides occurs in low- and middle-income countries. It is predicted that, by 2020, an average of one suicide death occurs by every 20s and one suicide attempt occurs per every 1 or 2s [3, >^4^].

Estimates indicate that the number of suicide cases are under-reported. In most countries of the world, a death is recorded as a suicide when natural causes of death, accident or murder are rejected or there is a note indicating the act of suicide by the deceased person. Some suicide cases are registered as death by accident because it was not possible to rule out other causes of death and accident, in particular. Reasons such as religion, ethical cultural factors, socio-political factors, and faults in issuing death certificates present a challenge in the manner of death registration and lead to UR. Suicide statistics in most countries are under-reported. Size of UR may vary from country to country and even from town to town. In general, the size of UR in developing countries is increasing [1, 5]. Suicide is a complex phenomenon that may occur over time and based on individual interaction with different risk factors [6]. Suicide prevention requires careful planning. World Health Organization considers suicide as a preventable cause of death. Studies conducted in various countries proved the effectiveness of interventions [7, >^8^]. The first step for implementation of any intervention and prevention program is to have precise statistics of the current status of suicide [9].

 Various methods have been developed to estimate the size of hardly accessible populations; one of these methods is Network Scale-up (NSU).  NSU estimates the size of target population using data retrieved from the general population [10]. Given that suicide is becoming one of the largest public health problem and also, there is no care system and detailed report in this regard [>^11^], this study was undertaken in order to estimate UR of suicides and SAS in Kermanshah Province which is among provinces with high suicide death and suicide attempt rates in Iran. 

## Materials and Method

 *The Study population*

 Present study is a cross-sectional research which is carried out in Kermanshah. With about 1-million populations, Kermanshah is the westernmost province in Iran which shares its borders with Iraq. For estimating the size of under-reported SAS and suicide death registers, all cases were retrieved from forensic medicine and health centers, in 2014. Then, using network scale up method, a sample of 500 cases, aged 18 to 65 years, were randomly selected from the general population on the basis of age - sex proportion of. To find the 95% confidence interval, bootstrap technique was used. In Iran, death records cover only two suicide methods: self-immolation and deliberate self-poisoning. There is no record for other methods of suicide. 

 *Data collection*

 Forensic medicine staff report suicide death registers information, such as demographic and time data, cause and location of the incident, and other information on a daily basis after interviewing suicide attempters’ relatives and collect document and medical records on a monthly basis. Information about the self-immolation and deliberate self-poisoning were only collected from poisoning and burns treatment centers. More than 90% of (non-) deliberate self-poisoning cases as well as about 100% of burning cases, who need treatment, refer to this center. In these centers, psychology experts are stationed to help recording information on suicide including: demographic data, cause of suicide attempt, time of the accident and psychological status of the attempters. In the first place, using experts’ idea, different areas of Kermanshah were divided to three - high, medium and low - socio-economic categories. Then, cluster sampling was performed and, after consulting with experts, 5 highly commuted clusters were assigned to each category. To increase participation and information accuracy, added to employing both female and male interviewers, they were trained role-play skill and interviewed the study subjects of their same sex. Inquiry in the considered areas of study was conducted in two sessions; morning and evening.   

 *NSU Methodology*

  Introduced for the first time in 1986, NSU is a network expansion method which estimates the size hidden population. NSU is based on social network structure theory and interviews random samples of the target population. NSU does not ask about the interviewee yet asks how many people he/she is connected to. So, first you need to know to how many people the respondents are connected, on average. The size of the respondent’s social network is assumed to be (c). The estimated size of active network of Iranian population was 308 [12]. According to NSU standards, in the current study, Acquaintances refers those whom the respondents knew by their names and/or faces, met them at least once during 2 past years, and/or had telephone or e-mail contacts with them [10].

 *Inclusion and exclusion criteria*

 NSU-based inclusion criteria were: being at age range between 18 and 65, being resident of the study areas during the last year, being able to have aural and verbal communication with the interviewers, and not having consciousness problem. Exclusion criteria were not being resident of Kermanshah and refusing informed consent to participate in the study. Given that health centers in Kermanshah are referral centers in the West of Iran, deaths and suicides referred from other cities and provinces were excluded.

 *Ethics*

 This study was conducted based on Helsinki Declaration. There was no need to take consent of people for already available data at forensic medicine and medical centers, but to increase ethics, the principles and rules of confidentiality were observed. NSU-based data collection was performed without registering interviewees’ name or last name and after getting their verbal consent. All stages of the study design and implementation were approved by Ethical Committee of Kerman University of Medical Sciences (IR. KMU.REC. 2015. 440).

 *Data Analysis*

 In addition to describing data by descriptive indicators, data coverage rate was estimated using NSU method by dividing the number of registered cases to the number of estimated ones. *Relied on *random sampling method, the* confidence interval* was estimated based on *1000 bootstrap replicates. All d*ata analyses were performed using STATA software (Version 13; Stat Corp, College Station, Texas, USA).

## Results

From a total of 530 subjects, 30 were not willing to participate in the study. The response rate was 94.3%. Among the 500 respondents, there were 251 (50.2%) males and 249 (49.8%) females. 12 respondents (2.4%) had a history of attempted suicide. The average numbers of attempted suicides and suicides deaths that the respondents knew in their lifetime were about 1.35 and 1.38, respectively (Table 1). 

**Table 1:** Demographic Characteristics of Respondents of Subjects on the Number of Reported suicide

**Reported number of suicides per each interview during lifetime**		**No, of Interviews (%)**	**Determinants**	
**Suicide Attempts**	**Suicide Deaths**			
1.36	1.53	251(50.2)	Male	**Sex**
1.35	1.24	249(49.8)	Female	
1.61	1.42	43(8.6)	20 years ≥	**Age group**
1.21	1.09	131(26.1)	21-25 years	
1.25	1.66	99(19.8)	26-30 years	
1.46	1.36	119(23.8)	31-40years	
1.41	1.90	108(21.6)	41 years ≤	
1.10	1.00	46(9.2)	4 ≥	**Educational level** **(year)**
1.28	1.27	58(11.6)	5-8	
1.35	1.23	165(33.1)	9-12	
1.37	1.18	230(46.1)	13 ≤	
1.31	1.45	196(39.3)	Single	**Marital status**
1.39	1.29	303(60.7)	Married	
1.50	1.38	490(97.6)	No	**History of attempted suicide **
1.35	2.00	12(2.4)	Yes	

**Fig. 1:**
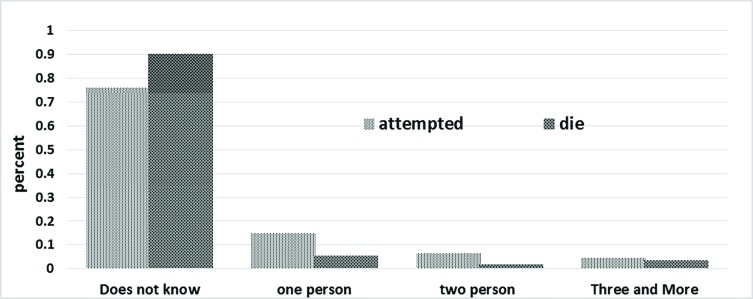
Number of suicide attempters known by respondents, based on result of suicide

The largest numbers of SDS and SAS that the respondents knew in social networks of their lifetime were 6 and 4, respectively. Generally, as indicated in the figure below, 379 (75.8%) and 453 (90.6%) respondents did not know any suicide attempter or suicide death during the last year of their life (Figure 1). 

Among suicide attempters known by respondents, 15 (11.9%) had not referred to hospital. Size estimation of SAS by NSU method revealed that deliberate self-poisoning with medication, poisoning with toxins and chemicals, and self-immolation were the most frequent methods of suicide, respectively. Finally, estimations showed that 61.7% of SAS in Kermanshah were self-poisoning with medication (Table 2). 

**Table 2:** Estimation of the annual number of fetal and non-fetal suicide based on methods of suicide

**NSU-based estimation** **by taking visibility rate into account**	**Visibility** **(%)**	**NSU-based estimation** **N (CI)a**	**Registered suicide deaths for one year **	**Reported suicides in 2015**	**Suicide attempts** **(** SAS**) **
**1595**	23.9	**381 **(308-454)	1315	57	**self-poisoning with medication, **
532	23.9	127 (74-174)	411	19	**poisoning with toxins and chemicals**
200	60.0	120 (73-168)	70	18	**Self-immolation **
54	85.0	46 (13-74)	----	7	**Hanging **
173	65.0	113 (61-147)	----	17	**Cutting objects and guns**
28	79.0	20 (7-40)	----	3	**Other b **
2584	---	809 (529-1090)	---	121	**Total**

In Kermanshah, death records cover only two suicide methods: self-immolation and deliberate self-poisoning. After taking the visibility rate into account and modifying the obtained value, average coverage of registrations for self-immolation and deliberate self-poisoning by toxins, medications, and chemicals were 82.4% and 77.2%, respectively. According to forensic medicine f Kermanshah, the number suicide death registers of 180. Hanging, self-immolation and deliberate self-poisoning were the most common methods of suicide. However, the number of suicide deaths estimated by NSU method (111-472) was 307. On average, 58.4% deaths registered as suicide were covered; the lowest and highest coverage rate of SDS were attributed to self-immolation (34.2%) and hanging (81.2%), respectively (Table 3). 

**Table 3:** Estimation of the number SDS using NSU method

**Covered registers**	**NSU-based estimation (95% CI)a**	**Registered number of suicide deaths for one year **	**Reported number suicide deaths in 2015 **	**Suicide deaths** **(** SDS**) **
44.8	87 (38-118)	39	12	**self-poisoning with medication, **
75.7	33 (3-53)	25	5	**poisoning with toxins and chemicals **
34.2	73 (32-108)	25	11	**Self-immolation **
81.2	73 (32-108)	65	11	**Hanging **
80.2	26 (6-53)	21	3	**Cutting objects and guns**
38.4	13 (0-32)	5	2	**Other b **
58.4	307 (111-472)	180	44	**Total**

a 95% confidence interval estimated by bootstrap technique; b Other suicide methods include: drowning, falling from height, electrocution

**Fig. 2:**
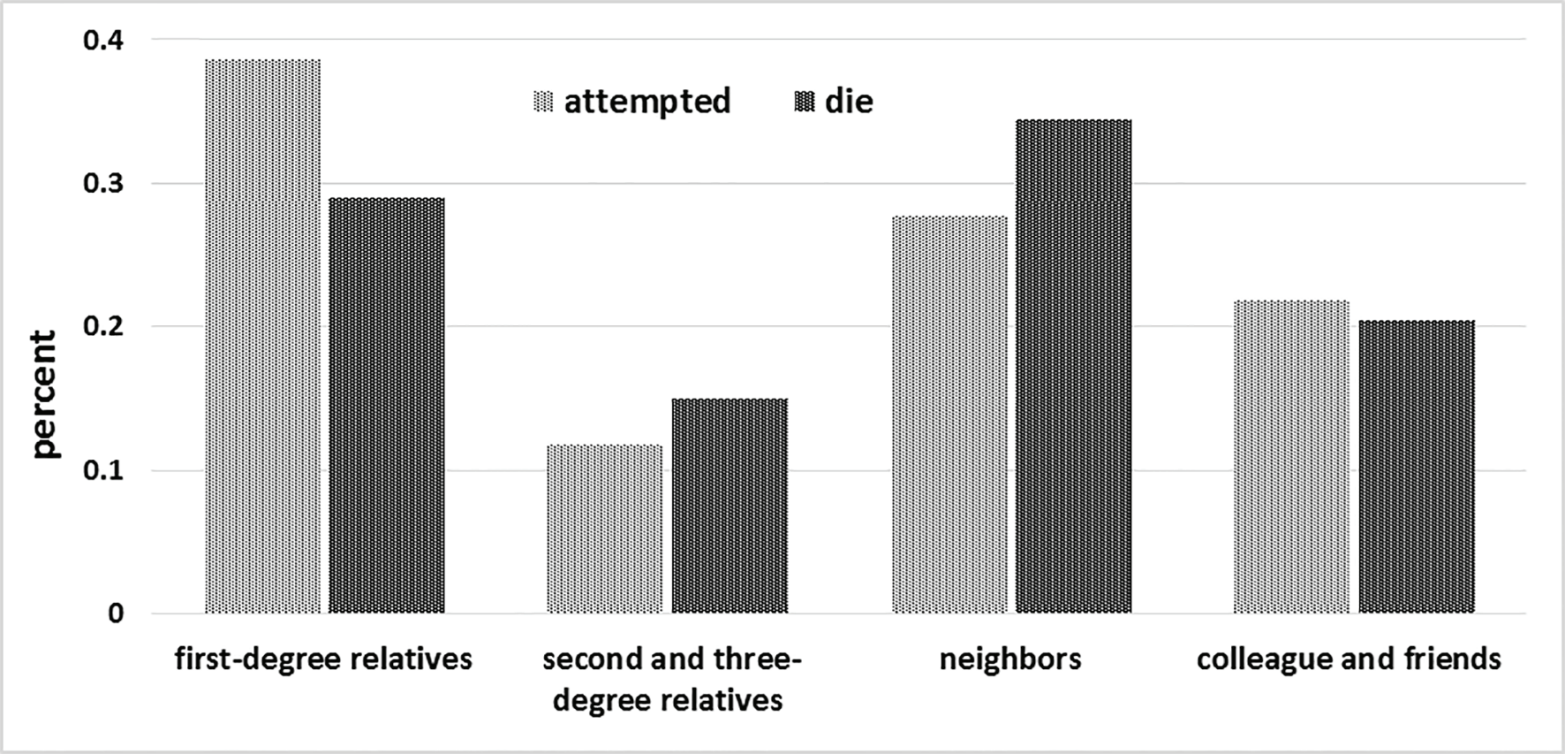
Degree of respondents’ connection to suicide attempters based on the result of suicide

The findings of this study showed a significant relationship between result of suicide and degree of connection to the respondents (*p*<0.001) so that most of the SDS and SAS occurred among neighbors and first-degree relatives of the respondents (Figure 2). 

## Discussion

Estimation of the number of suicides can help policymakers and authorities in taking preventive measures and providing appropriate services for those in need. Suicide is among the major causes of death which has been under-reported. Misclassification and strong social stigma attached to suicide are among the most significant reasons for UR of SDS[13]. Most of the SDS are registered under various names such as accidental drowning, accidental burning, accidental poisoning, road traffic accident, and natural deaths. Studies in Sweden, which has one of the most accurate and regulated death registration system, have shown that about 3% of road traffic accident deaths, 6% of natural deaths, and 21% of undetermined category deaths have to be registered as SDS[>^14^]. In France the situation is even worse such that 35% of deaths with undetermined category and 25% of deaths with unknown causes have to be registered as SDS[15]. The level of misclassification is different in each country. Due to misclassification, there are 65%, 32% and 35% under-reported suicides in England, Finland, and France, respectively. Overall under-reported cases of suicides in different countries ranges from 10 to 60% [14].

In 2014, the number suicide death registered in Kermanshah was 180; however, the number of SDS estimated by NSU method was 307; i.e. 58% of death registers were covered. Given the low level of suicide register coverage in Iran, all deaths with unclear causes or registered accidental deaths have to be reconsidered. Investigations of authenticity of causes of death and correction of wrong codes can greatly reduce UR of suicide in Iran. According to the World Health Organization, 20 SAS occur per each suicide death; however, based on studies conducted in Iran, the proportion of suicide death to suicide attempt is about 10% [16, >^17^]. Two main reasons are presented for this low proportion in Iran: 1) high fatality rate of suicide in Iran because of using violent methods with high rate of mortality such as self-immolation and hanging while the former is most common method among women and the latter is most common among men; 2) higher proportion of suicide attempt denial than suicide death. According to experts and the findings of this study, between 3000 and 5000 cases of attempted suicide have to be registered. Hence, with regard to existing literature and the current research findings, it can be concluded that NSU is a good estimator for SDS but it is not a suitable method for estimating the size of SAS. One main reason for such conclusion is low visibility and confidence level of NSU.  In general, given the suicide trend over time, it can be said that suicide data have poor validity but an acceptable level of reliability [>^18^, >^19^].

UR rate was the highest in younger age groups. In a similar study, the rate of UR decreased with increasing age which is consistent with findings of the current research [18].On one hand, since UR reaches its highest rate in 15-23 age-group, Iran has the largest population in this age group, and this group has the highest rate of SDS, and on the other hand, due to not so accurate death registration system in Iran, it is likely that mortality rate of suicide is much more than available records.

In the current research, less than 3% of the subjects had a history of suicide attempt.  The obtained rates for history of suicide attempt were different in similar studies. The most significant reasons for this difference in the studied population (general population, hospitalized population, and suicide attempters) were rate of suicide in the given society and fatality rate of suicide. In general, according to reports, history of suicide attempt among general population and suicide attempters varies from 1 to 5% and 3-20%, respectively. Size of UR varies for different suicide methods. UR of suicides which are classified as accidental death is very high. High rate of under-reported self-immolation may also be due to the same reason. Low rate of UR and register coverage for suicides that cannot be classified as accidental death may be due fault in registration systems.    

 *Strengths and Weaknesses*

 Size estimation of suicides using NSU method had several limitations, the most notable of which is uncertainty of respondents about confidentiality of data. Since the respondents were selected from the whole area of the city and they were interviewed in the same place, they could not completely trust the interviewers. The place for interview was not quite enough to answer sensitive questions. But, to lessen the level of mistrust, interviewers with the same sax of the respondents were selected and indirect questions were used. On the other hand, the present study had a lot of strengths including selection of a sample representing different areas of Kermanshah province, recruitment of trained staff, and appropriate sample size.

 In conclusion, although the rate of 6 per 100.000 people for suicide in Iran, reported by governmental organizations, is far from global average of 16 per 100.000, it should be noted that suicide death registers coverage is nearly 60% and there is not much difference between SD in Iran and global average. Given the low coverage of suicide registers in Iran, all causes of deaths, especially those with undetermined intent or deaths classified as accident, have to be reconsidered. Investigations of authenticity of causes of death and correction of wrong codes can greatly reduce UR of suicide in Iran. In addition, interview with survivors to give them assurance can reduce the rate of suicide denial and result in increased accuracy of death register coverage. 

## Funding:

None

## Conflicting interests:

Authors have no conflicting interests to disclose.

## Ethical approval:

All stages of the study design and implementation were approved by Ethical committee of Kerman University of Medical Sciences (IR. KMU.REC. 2015. 440).
